# Balancing sensory inputs: somatosensory reweighting from proprioception to tactile sensation in maintaining postural stability among older adults with sensory deficits

**DOI:** 10.3389/fpubh.2023.1165010

**Published:** 2023-05-04

**Authors:** Ziyin Liu, Qi Wang, Wei Sun, Qipeng Song

**Affiliations:** College of Sports and Health, Shandong Sport University, Jinan, China

**Keywords:** sensory reweighting, biomechanics, peripheral neuropathy, postural control, rehabilitation

## Abstract

**Background:**

Sensory deficits increase the risk of falls among older adults. The purpose of this study was to investigate the correlations of lower extremity muscle strength, proprioception, and tactile sensation to postural stability among older adults with and without sensory deficits, to understand the contribution of each factor to postural stability, and to explore sensory reweighting among the two populations.

**Methods:**

A total of 103 participants were recruited and divided into two older adult groups with (female = 24, male = 26, age = 69.1 ± 3.15 years, height = 162.72 ± 6.94 cm, body mass = 64.05 ± 9.82 kg) and without sensory deficits (female = 26, male = 27, age = 70.02 ± 4.9 years, height = 163.76 ± 7.60 cm, body mass = 65.83 ± 10.31 kg), based on whether a 5.07 Semmes–Weinstein monofilament could be detected at foot soles. Their Berg Balance Scale (BBS), lower extremity muscle strength, proprioception, and tactile sensation were tested and compared between the two groups. Pearson's or Spearman's correlations were used to explore the relationships between the BBS and each variable. Factor analysis and multivariate linear regression were used to verify the degrees of correlation between the generated factors and the postural stability.

**Results:**

Low BBS (*p* = 0.003, η^2^ = 0.088) scores and higher proprioception thresholds (knee flexion: *p* = 0.015, η^2^ = 0.059; knee extension: *p* = 0.011, η^2^ = 0.065; ankle plantarflexion: *p* = 0.006, η^2^ = 0.075; ankle dorsiflexion: *p* = 0.001, η^2^ = 0.106) were detected among older adults with sensory deficits compared with those without sensory deficits. Lower extremity muscle strength (ankle plantarflexion: *r* = 0.342, *p* = 0.002; hip abduction: *r* = 0.303, *p* = 0.041) and proprioception (knee flexion: *r* = −0.419, *p* = 0.004; knee extension: *r* = −0.292, *p* = 0.049; ankle plantarflexion: *r* = −0.450, *p* = 0.002; ankle dorsiflexion: *r* = −0.441, *p* = 0.002) were correlated with BBS among older adults without sensory deficits, while lower extremity muscle strength (ankle plantarflexion: *r* = 0.501, p<0.001; hip abduction: *r* = 0.302, *p* = 0.041) and tactile sensation (great toe: *r* = −0.388, *p* = 0.008; 5th metatarsal: *r* = −0.301, *p* = 0.042) were correlated with BBS among older adults with sensory deficits.

**Conclusion:**

Older adults with sensory deficits have poorer proprioception and postural stability. Somatosensory reweighting occurs from proprioception to tactile sensation among older adults with sensory deficits in maintaining postural stability.

## 1. Introduction

More than 15% of older adults over the age of 60 have sensory deficits (1), and they are at higher risk of falls (2), which is one of the significant risk factors for injury and even death (3). Falls result in minor injuries (28%), soft tissue injuries (11%), and fractures (5%) (4), and 20% of those who fall require long-term medical care (4, 5). In China, falls cost $743–$3,742 per older adult (6).

Deficient postural stability, commonly measured by the Berg Balance Scale (BBS) (7), is one of the most substantial risk factors for falls (8). The maintenance of postural stability requires the integration of sensory inputs and neuromuscular control (9). Lower extremity muscle strength, proprioception, and tactile sensation are three potential factors for maintaining postural stability and preventing falls in daily activities (7, 10). Adequate lower extremity muscle strength is necessary to generate corrective torques during the perturbations (10). Ankle and hip strength are closely related to postural stability since ankle and hip strategies are commonly adopted when body balance is disturbed (11, 12). In addition, as the main elements of somatosensory feedback, proprioception and tactile sensation percept one's body and movements inside the body and physical characteristics of the environment outside the body (13). Signals from tactile afferents and proprioceptive evoke coordinated motor patterns, which rapidly modify the locomotor pattern in response to perturbations or unexpected environmental changes (13).

It has been suggested that sensory deficits are strongly associated with the inability to perceive 5.07 Semmes–Weinstein monofilament (SWM) (14) and decreased light touch sensation due to small neurofibrillary lesions can be detected by SWM tests (14). Only a few studies investigated whether proprioception deteriorates among individuals with sensory deficits, and it is controversial whether lower extremity muscle strength and postural stability were worse. One study indicated proprioception decreased as individuals temporarily reduced tactile sensation by anesthesia (15). Another argued that tactile deficits caused by anesthesia differ from typical tactile deficits due to neuroplasticity and sensory reweighting (16).

The correlations of postural stability to lower extremity muscle strength, proprioception, and tactile sensation among older adults with sensory deficits are inconclusive. Several studies indicated that lower extremity muscle strength is related to postural control among individuals with sensory deficits (17, 18). Still, no studies compared the correlations between lower extremity muscle strength and postural stability among individuals with and without sensory deficits. Some studies pointed out that proprioception is crucial for postural stability (19, 20). However, one study indicated that proprioception is unrelated to postural stability among individuals with sensory deficits (18). One study detected a significant correlation between tactile sensation and postural stability among individuals with sensory deficits (21), while another showed no correlation (22).

When the body receives less or inaccurate sensory information, the central nervous system dynamically assigns different weights to multiple available sensory cues (23), thus reducing the influence of unreliable sensory cues and increasing the influence of other sensory cues that provide more reliable information (24); this process is called sensory reweighting (25). Proprioception and tactile sensation deteriorations have been observed among older adults with sensory deficits (2, 26); however, to our knowledge, no studies have investigated sensory reweighting among this population.

Determining the lower extremity muscle strength and sensation characteristics, clarifying the relationship of lower extremity muscle strength, proprioception, and tactile sensation to postural stability, and identifying the presence of sensory reweighting among older adults with sensory deficits can facilitate the development of targeted rehabilitation programs for this fall-prone population. It is hypothesized that (1). compared to older adults without sensory deficits, lower extremity muscle strength, worse proprioception, and poorer postural stability would be detected among older adults with sensory deficits; (2). significant correlations of postural stability to lower extremity muscle strength, proprioception, and tactile sensation would be detected among older adults with and without sensory deficits; (3). strength would have the strongest correlations with postural stability, followed by proprioception and tactile sensation; and (4). sensory reweighting occurs between tactile sensation and proprioception among older adults with sensory deficits.

## 2. Methods

### 2.1. Participants

An *a priori* power analysis (G^*^ power version 3.1) showed that a minimum of 39 participants would be required in each group to achieve an alpha level of 0.05 and a statistical power of 0.80 based on the previous report (27), which detected *r*^2^ = 0.316 between tactile sensation and postural stability in 95 older adults. The participants were enrolled by distributing flyers and presentations in local communities and nursing homes. Inclusion criteria were as follows: (1) 65 years of age or older; (2) independent ambulation without assistive devices; and 93) no cognitive impairment as defined by the Brief Mental State Examination score>24. Exclusion criteria included the following: (1) Self-reported history of central nervous system dysfunction; (2) deficits in visual function, dizziness, vertigo, or any other vestibular disorder; (3) psychological problems associated with falls, such as fear of falling, anxiety, or depression; and (4) evidence of sole foot ulcers by direct assessment. A total of 103 participants were recruited for this study. A total of 50 participants who could not detect a 5.07 SWM on any of the plantar positions were enrolled in the sensory deficits group (28) (female = 24 and male = 26, age = 69.1 ± 3.15 years, height = 162.72 ± 6.94 cm, body mass = 64.05 ± 9.82 kg), and 53 gender- and age-matched participants without sensory deficits were enrolled in the control group (female = 26 and male = 27, age = 70.02 ± 4.9 years, height = 163.76 ± 7.60 cm, body mass = 65.83 ± 10.31 kg). No significant differences were found between the two groups in age, height, and weight using independent-samples *t*-tests. All the participants signed informed consent forms before the formal tests. The project was approved by the Ethics Committee of Shandong Sports University (19003) following the Declaration of Helsinki.

### 2.2. Protocol

The BBS, lower extremity muscle strength, proprioception, and tactile sensation were measured for all eligible participants. The order of the BBS, proprioceptive, and tactile sensation tests was randomized, and the lower extremity muscle strength was tested last to avoid fatigue. The data for this study were collected in Jinan City of Shandong Province, China, from July to September 2019.

### 2.3. BBS test

The BBS test consists of 14 simple tests of daily functional activities (e.g., get up from a sitting position and turn 360°) with good inter-rater and intra-rater reliability (ICC = 0.98–0.99) (29). A score of 0–4 was awarded based on the participant's performance during each test, and the scores were accumulated when the 14 tests were completed.

### 2.4. Lower extremity muscle strength test

**Figure 1:**
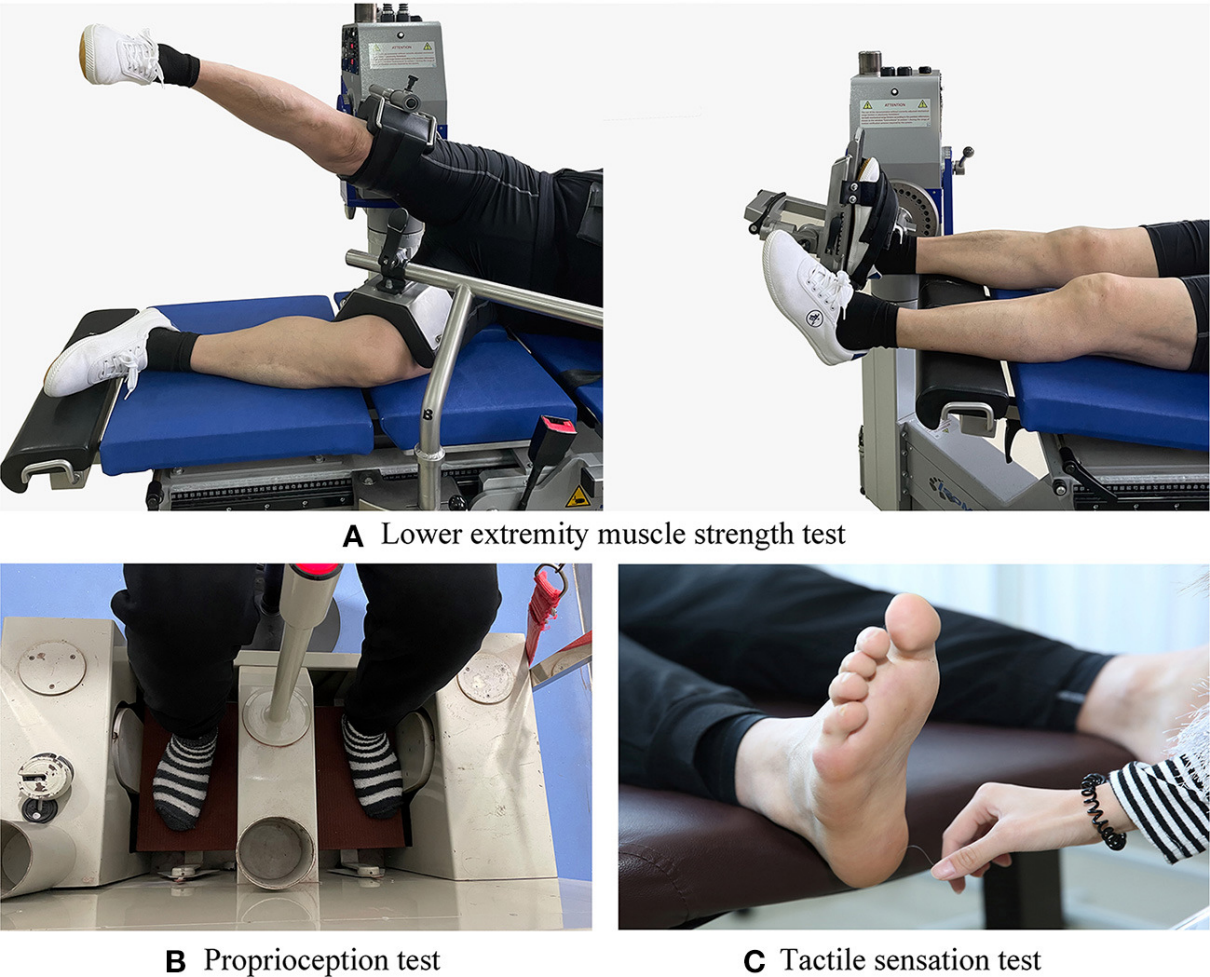
Test illustrations: **(A)** Lower extremity muscle strength test. **(B)** Proprioception test. **(C)** Tactile sensation test.

The IsoMed 2000 strength testing system (D. & R. Ferstl GmbH, Hemau, Germany) with good test–retest reliability (ICC value, 0.77–0.98) (30) was used to measure participants' maximum isokinetic joint torque of ankle plantar/dorsiflexion and hip abduction of their dominant side at an angular velocity of 60°/s (Figure 1A). During the ankle muscle strength test, each participant lay supine on a plyometric bed, keeping their hips and knees extended. His/her back, thigh, and foot of the test side were tied up with straps and secured with Velcro to ensure ankle joint stability. Ankle plantarflexion started at 5° of dorsiflexion and stopped at 30° of plantarflexion, while dorsiflexion started at 30° of plantarflexion and stopped at 5° of dorsiflexion. In the hip muscle strength test, each participant lay on their side with their pelvis and leg of the test side secured with straps. Hip abduction started from 0° of hip abduction and stopped at 30° of hip abduction. A total of three tests were conducted in each direction, and the mean values were taken for data analysis (10).

### 2.5. Proprioception test

The proprioception thresholds for the ankle and knee on each participant's dominant side were assessed using a proprioception test device (Figure 1B), which showed good test–retest reliability [intraclass correlation coefficient (ICC) = 0.74–0.94] (31). The proprioception test device collected the minimum angular motion the patient can detect during knee flexion/extension and ankle dorsal/plantarflexion. The device consists of a box and a platform that can rotate within the frontal and sagittal planes. Two electric motors drive the platform at an angular velocity of 0.4°/s. The movement of the platform can be stopped at any time by a hand switch controlled by the participant. Each participant was seated on a height-adjustable chair with their foot on the platform. During the ankle proprioception test, the knee and hip joints were flexed at 90°, and the leg was perpendicular to the surface of the platform when the platform was placed in a horizontal position. During the knee proprioception test, the lateral axis of the instrumentation was parallel to the mediolateral axis of the knee joint. The hip and knee joints were each positioned at 90°, and the ankle joint was in a neutral position. Approximately 50% of the weight of the participant's lower extremity was resting on the platform, and a thigh cuff suspension system was used to control unwanted sensory cues from contact between the platform and the plantar surface of the foot. Each participant was instructed to concentrate on their foot and to press the hand switch to stop the movement of the platform when they could sense motion; they were then asked to identify the direction of rotation. The motor was equipped to rotate with a random time interval ranging from 2 s to 10 s after the indication to start a trial. At least five tests were conducted in each direction, and the mean values were taken for data analysis (7).

### 2.6. Tactile sensation test

The tactile sensation was tested using a set of SWM (North Coast Medical, Inc., Morgan Hill, CA, USA) (Figure 1C), which showed good test–retest reliability (ICC values, 0.83–0.86) (32). Monofilaments of six different sizes were used in this study: 2.83, 3.61, 4.31, 4.56, 5.07, and 6.65; each of them applies 0.07 g, 0.40 g, 2.00 g, 4.00 g, 10.00 g, and 300.00 g of force when pressed into a C-shape (bent 90°). The tests were performed by randomly stimulating the dominant foot sole of the big toe, first and fifth metatarsal heads, arch, and heel with filaments from thinnest to the thickest. The duration of each stimulation was ~1 s, and the stimulus was repeated twice for each site. The sensitivity threshold for each site was determined by the thinnest monofilament the participant could feel. The lower the sensitivity threshold, the better the plantar tactile sensation.

### 2.7. Statistics

Shapiro–Wilk test was used to test whether the data were normally distributed. Group differences were tested by independent-samples *t*-tests (normal distribution) or the Mann–Whitney U-tests (non-normal distribution), and Cohen's *d* or η^2^ was used as their effect sizes, respectively. The thresholds for Cohen's *d* were as follows: < 0.20, trivial; 0.21–0.50, small; 0.51–0.80, medium; >0.81, large. The thresholds for η^2^ were as follows: 0.01–0.059, small; 0.06–0.14, medium; >0.14, large.

Pearson's (normal distribution) or Spearman's (non-normal distribution) correlations were used to determine the correlations of the BBS score with each of the lower extremity muscle strength, proprioception, and tactile sensation variables while controlling for the covariates, age, height, and weight. The thresholds for the correlation coefficient (r) were as follows: 0–0.1, trivial; 0.1–0.3, weak; 0.3–0.5, moderate; >0.5, strong (33). A separate exploratory factor analysis was carried out among each category of the variables of interest. Multivariable linear regression was used to explore the relationship between each generated factor and outcome while controlling for the other covariates. All analyses were conducted in SAS 9.4, and the significance level was set at 0.05.

## 3. Result

The Shapiro–Wilk tests showed that the BBS, proprioception, and tactile sensation data were non-normally distributed, and the lower extremity muscle strength data were normally distributed.

The descriptive characteristics of lower extremity muscle strength, proprioception, and tactile sensation are shown in Table 1. Significant between-group differences were detected in BBS, proprioception, and tactile sensation, while not in strength.

**Table 1:** Lower extremity muscle strength, proprioception, and tactile sensation in sensory deficits and control groups.

		**Sensory deficits**	**Control**	***p***	***d***	**η^2^**
BBS	**–**	52.3 ± 2.68	53.8 ± 2.41	**0.003**	–	0.088
Lower extremity muscle strength (*N* * m/kg)	Ankle plantarflexion	0.35 ± 0.19	0.38 ± 0.14	0.356	0.180	–
	Ankle dorsiflexion	0.21 ± 0.07	0.22 ± 0.06	0.640	0.093	–
	Hip abduction	0.40 ± 0.18	0.45 ± 0.15	0.113	0.317	–
Proprioception (°)	Knee flexion	3.17 ± 2.18	2.59 ± 1.93	**0.015**	–	0.059
	Knee extension	3.71 ± 2.76	2.76 ± 1.90	**0.011**	–	0.065
	Ankle plantarflexion	5.06 ± 4.25	2.99 ± 2.62	**0.006**	–	0.075
	Ankle dorsiflexion	4.36 ± 3.51	2.57 ± 2.08	**0.001**	–	0.106
Tactile sensation (gauge)	Great toe	4.55 ± 0.79	4.14 ± 0.38	**< 0.001**	–	0.133
	1st Metatarsal	4.58 ± 0.78	4.10 ± 0.38	**< 0.001**	–	0.115
	5th Metatarsal	4.63 ± 0.64	4.18 ± 0.35	**< 0.001**	–	0.226
	Arch	4.79 ± 0.75	4.24 ± 0.33	**< 0.001**	–	0.242
	Heel	5.11 ± 0.82	4.35 ± 0.29	**< 0.001**	–	0.310

Data are presented as mean ± SD.

Bold: Significant difference between the sensory deficits and control groups.

Correlations of BBS score to lower extremity muscle strength, proprioception, and tactile sensation variables are shown in Table 2. In the sensory deficits group, the BBS was moderately to strongly correlated with the muscle strength of ankle plantarflexion and hip abduction and moderately correlated with the tactile sensation of the great toe and first metatarsal head. In the control group, the BBS was moderately correlated with muscle strength of ankle plantarflexion and hip abduction and weakly to moderately correlated with proprioception of knee flexion/extension and ankle plantar/dorsiflexion.

**Table 2:** Correlations of BBS score to lower extremity muscle strength, proprioception, and tactile sensation variables.

**Variables**		**Sensory deficits**		**Control**	
		***r***	***p***	***r***	***p***
Lower extremity muscle strength (*N* * m/kg)	Ankle plantarflexion	0.501	**< 0.001**	0.342	**0.002**
	Ankle dorsiflexion	0.244	0.102	0.042	0.784
	Hip abduction	0.302	**0.041**	0.303	**0.041**
Proprioception (°)	Knee flexion	−0.245	0.101	−0.419	**0.004**
	Knee extension	−0.125	0.408	−0.292	**0.049**
	Ankle plantarflexion	−0.243	0.104	−0.450	**0.002**
	Ankle dorsiflexion	−0.264	0.076	−0.441	**0.002**
Tactile sensation (gauge)	Great toe	−0.388	**0.008**	−0.113	0.456
	1st Metatarsal	−0.072	0.635	0.163	0.279
	5th Metatarsal	−0.301	**0.042**	0.230	0.124
	Arch	−0.163	0.280	−0.004	0.977
	Heel	−0.115	0.447	0.020	0.896

BBS, Berg Balance Scale.

Bold: Significant correlations.

Adjusted for age, weight, and height.

Factor loadings of lower extremity muscle strength, proprioception, and tactile sensation in sensory deficits and control groups are shown in Table 3. In both groups, factor 1 (F1), factor 2 (F2), and factor 3 (F3) are the sum of lower extremity muscle strength, proprioception, and tactile sensation, respectively.

**Table 3:** Factor loadings of lower extremity muscle strength, proprioception, and tactile sensation in sensory deficits and control groups.

		**Factor loading (Sensory deficits)**			**Factor loading (Control)**		
		**F1**	**F2**	**F3**	**F1**	**F2**	**F3**
Lower extremity muscle strength	Ankle plantarflexion	0.904	–	–	0.820	–	–
	Ankle dorsiflexion	0.891	–	–	0.717	–	–
	Hip abduction	0.892	–	–	0.702	–	–
Proprioception	Knee flexion	–	0.800	–	–	0.880	–
	Knee extension	–	0.788	–	–	0.813	–
	Ankle plantarflexion	–	0.865	–	–	0.848	–
	Ankle dorsiflexion	–	0.755	–	–	0.925	–
Tactile sensation	Great toe	–	–	0.763	–	–	0.718
	1st Metatarsal	–	–	0.690	–	–	0.706
	5th Metatarsal	–	–	0.716	–	–	0.629
	Arch	–	–	0.679	–	–	0.760
	Heel	–	–	0.774	–	–	0.736

Factor loading (Sensory deficits).

F, Factor.

–: Factor loading < 0.5.

The equations for multivariable regression are as follows:


(1) $$\begin{array}{l} BBS\;\left(sensorydeficits\right)=53.820+0.585\times F1-1.208\times F2 \end{array}$$

(2) $$\begin{array}{l} BBS\;\left(control\right)=52.300+1.531\times F1-0.703\times F3 \end{array}$$

In equation 1, variance inflation factor (VIF) = 1.221, adjusted *r*^2^ = 0.522, p_F1_ = 0.045, p_F2_ < 0.001, β_F1_ = 0.242, and β_F2_ = −0.500. In equation 2, VIF = 1.157, adjusted *r*^2^ = 0.683, p_F1_ < 0.001, p_F3_ = 0.019, β_F1_ = 0.571, and β_F3_ = −0.262.

F3 from equation 1 and F2 from equation 2 were excluded as their P-values were > 0.05. The equations indicated that in the sensory deficits group, proprioception has contributed more to BBS than lower extremity muscle strength (β_F2_ > β_F1_), while in the control group, lower extremity muscle strength has contributed more to BBS than tactile sensation (β_F1_ > β_F3_).

## 4. Discussion

This study compared postural stability and its three potential factors, namely lower extremity muscle strength, proprioception, and tactile sensation among older adults with and without sensory deficits, and investigated the relationship between these three factors and postural stability in the two groups. The findings partly supported hypotheses #1 and 2 and rejected hypotheses #3 and 4.

Poorer postural stability and worse proprioception were detected among older adults with sensory deficits, compared with those without sensory deficits. The lower BBS score is consistent with previous studies (2, 26). Much evidence indicates that tactile sensation is critical for postural stability (26). When balance is disturbed, the deteriorated tactile sensation cannot accurately detect the contact between the foot and the ground, making it difficult to control the transfer of body weight caused by the disturbance (34). The worse proprioception is consistent with some previous studies (28, 35). Proprioception and tactile sensitivity are important components of the somatosensory system (16), and both work in synergy and are interconnected through interneurons and alpha neurons (36). The connection of proprioception and tactile sensation may line in the sharing receptors; e.g., the rapidly adapting fiber receptors from Pacinian corpuscles, which are typical tactile receptors, make an essential contribution to proprioception (37).

Significant correlations of lower extremity muscle strength to postural stability were detected in both groups, which is consistent with some previous studies (7, 10) and inconsistent with another one (38), in which there was no correlation of ankle strength to postural stability was detected. However, only static postural stability was tested in their study, and postural control included static balance and dynamic balance (9). BBS test comprises the above two balance tasks to predict fall risk by evaluating the overall balance performance under different tasks to reflect the postural control ability of the elderly in daily activities. As lower extremity muscle strength was significantly correlated with postural stability in both groups in our study, strength-goal rehabilitation may enhance postural stability among older adults with and without sensory deficits.

In the control group, postural control was related to proprioception but not tactile sensation. The priority of proprioception over tactile sensation for postural control was supported by many studies (7, 10). However, one study challenged us by showing a significant correlation between tactile sensation and postural control (21). The disagreement may line in the participants; we recruited participants with normal tactile sensations in our control group, while they combined participants with normal and mild deficit tactile sensations (21). Peripheral sensory signals are transmitted by different sensory neurons (16). The type I and II sensory nerves, which transmit proprioception, are larger in diameter and conduct faster than the type III sensory nerves, which transmit tactile sensations (16), and the central nervous system usually relies more on proprioception than tactile sensation to facilitate posture maintenance (10).

Among older adults with sensory deficits, postural control was related to tactile sensation but not proprioception. Humans integrate signals from different sensory systems, with the weight of each signal being proportional to the relative reliability of the signal, with less reliable signals being given less weight (39). Sensory reweighting has been detected in patients with neurological disorders, e.g., stroke patients increasingly rely on vision to maintain balance after their somatic sensation is reduced (40). In this study, somatosensory reweighting from proprioception to tactile sensation seems to have occurred among older adults with sensory deficits. Our outcomes indicated that older adults in the sensory deficits group showed deficits in proprioception, and the unreliability of proprioceptive afferent information led them to rely more on tactile sensation. Similar findings showing the changed contribution of proprioception to postural stability have been observed. Zhang et al. detected a significant correlation between the H-index (represents the arc of the type I reflex loop, responsible for proprioception transmission) and postural stability in those with peripheral neuropathy but not in the controls that exhibited normal tactile sensation (16). It is difficult to explain the mechanisms behind our observations, which may relate to the function of the cortex (41). In a study of chronic low back pain, diffuse and non-specific changes in functional connectivity between brain regions have been observed, affecting the cortex function of proprioceptive regions (41). It can be inferred that sensory deficits would decrease the function of the proprioceptive cortex, which further down-weights the proprioceptive afferents.

We have previously investigated the correlation of lower extremity muscle strength, proprioception, and tactile sensation to postural stability in pooled older adults with and without sensory deficits, and the findings were similar to those of the control group (10). This is not surprising since only about 16% (27 of 163) of the older adults in the previous study had sensory deficits. The participants in this study overlapped with the previous one, with about half of older adults with sensory deficits being newly recruited and those without sensory deficits being matched from the previous database. This study separated the two populations and made a novel finding that somatosensory reweighting occurred from proprioception to tactile sensation among older adults with sensory deficits, which may indicate that different rehabilitation strategies should be adopted among patients with sensory deficits than those with normal sensation.

## 5. Limitation

The study has some limitations. First, visual and vestibular sensations were not measured. They may involve in the process of sensory reweighting. However, only a minor contribution was made by the visual and vestibular senses (30%), compared with proprioception and tactile sensation (70%) (16). Second, all participants were recruited from the same region, they had similar backgrounds, and caution should be taken when applying our findings to other populations.

## 6. Conclusion

Older adults with sensory deficits have poorer proprioception and deficit postural stability than those without sensory deficits; older adults without sensory deficits rely on lower extremity muscle strength and proprioception, while those with sensory deficits rely on lower extremity muscle strength and tactile sensation to maintain postural stability. Somatosensory weighting from proprioception to tactile sensation occurs among older adults with sensory deficits to maintain postural stability.

## Data availability statement

The raw data supporting the conclusions of this article will be made available by the authors, without undue reservation.

## Ethics statement

The studies involving human participants were reviewed and approved by the Ethics Committee of Shandong Sports University. The patients/participants provided their written informed consent to participate in this study.

## Author contributions

ZL participated in the design of the study and contributed to the collection and analysis of the data and drafted the manuscript. QW participated in the design of the study and data reduction/analysis. WS contributed to data collection and data analysis. QS participated in the design of the study and contributed to the interpretation of results and revision of the manuscript. All authors have read and approved the final version of the manuscript and agreed with the order of presentation of the authors.
